# Neobladder saving caesarean section in a pregnant woman with congenital bladder exstrophy

**DOI:** 10.1007/s00404-025-07937-4

**Published:** 2025-01-31

**Authors:** Chiara Granieri, Silvia D’Ippolito, Aniello Foresta, Benedetta Gui, Nazario Foschi, Giovanni Esposito, Giovanni Scambia

**Affiliations:** 1 Dipartimento di Scienze della Salute della Donna, del Bambino e di Sanità Pubblica, Fondazione Policlinico Universitario A Gemelli IRCCS, Largo A. Gemelli 8, 00168 Rome, Italy; 2 Dipartimento di Medicina e Scienze della Salute “Vincenzo Tiberio”, Università degli Studi del Molise, UNIMOL, Via Francesco De Sanctis 1, 86100 Campobasso, Italy; 3 Dipartimento di Diagnostica per Immagini, Radioterapia Oncologica ed Ematologia, Fondazione Policlinico Universitario Agostino Gemelli, IRCCS, 00168 Rome, Italy; 4 Urology Department, Fondazione Policlinico Universitario Agostino Gemelli, IRCCS, 00168 Rome, Italy; 5 Dipartimento di Scienze della Vita e Sanità Pubblica, Università Cattolica del Sacro Cuore, Rome, Italy

**Keywords:** Bladder exstrophy, Neobladder, Pregnancy management, Caesarean section

## Abstract

**Purpose:**

To propose an obstetric management model for patients with congenital bladder exstrophy (BE) and multiple previous surgeries, recognizing the unique anatomical variations of each patient and emphasizing the importance of personalized treatment approaches.

**Methods:**

We present the obstetric management and delivery of a 32-year-old pregnant woman with congenital BE, focusing on antenatal anatomical assessment, accurate delivery planning and key surgical steps during an elective caesarean section. Additionally, we conduct a comprehensive review of the current literature to enhance understanding of the reproductive implications associated with this exceptionally rare condition.

**Results:**

Following urological magnetic resonance imaging and intraoperative ultrasound, a successful neobladder-saving caesarean section was performed at 37 weeks, resulting in the delivery of a healthy infant with no maternal complications.

**Conclusion:**

Obstetric management in women with congenital BE presents unique challenges due to its significant impact on urinary and reproductive functions. Careful delivery planning and antenatal anatomical assessment are crucial for optimizing both maternal and fetal outcomes. A personalized, multidisciplinary approach can help minimize potential complications.

**Supplementary Information:**

The online version contains supplementary material available at 10.1007/s00404-025-07937-4.

## What does this study add to the clinical work

**Table:** 

This study demonstrates the feasibility of performing a neobladder-saving cesarean section in a pregnant woman with congenital bladder exstrophy. We highlight the importance of personalized surgical planning, multidisciplinary collaboration, and the use of antenatal and intraoperative imaging to optimize maternal and fetal outcomes.	

## Introduction

Bladder exstrophy (BE) is a rare congenital malformation with a prevalence of about 1 out of 50,000 live births [1]. This condition is part of the Exstrophy–Epispadias Complex (EEC), a rare spectrum of congenital defects affecting multiple systems, including the genitourinary and gastrointestinal tracts, musculoskeletal system, and pelvic muscles and bones. BE differs from cloacal exstrophy, a more severe birth anomaly that presents with various other defects, including malformations of the gastrointestinal and central nervous systems, collectively known as the OEIS (omphalocele, exstrophy, imperforate anus, spinal defects) complex [2]. Patients with BE are characterized by bladder eversion through the anterior abdominal wall, associated with separated pubic bones, a low-lying umbilicus, bifid clitoris and a short vagina. Advances in reconstructive surgery techniques and the use of antibiotic therapies have significantly improved both survival rates and quality of life in these patients [3–5]. BE repair typically involves several surgical procedures, including bladder and abdominal wall closure, ureteral reimplantation, and bladder neck reconstruction in early life. Even if bladder closure is successful, augmentation cystoplasty might be necessary. This procedure often utilizes segments of the bowel or ureter to expand the bladder wall. Following augmentation cystoplasty, continent urinary diversion is often required, utilizing a segment of colon or ileum to create a continent stoma for intermittent catheterization [2]. The introduction of Mitrofanoff technique, which creates a conduit connecting the neobladder to the skin, has enabled clean self-catheterization, thereby enhancing patients' ability to maintain continence [6]. Obstetric management in women with BE is particularly challenging due to its significant impact on urinary and reproductive functions. These patients often suffer from recurrent urinary infections, increasing the risk of premature delivery. Additionally, the osteoarticular anomalies associated with BE increase the likelihood of abnormal fetal presentation. A history of repeated corrective surgical procedures leads to multiple abdominal and pelvic adhesions. The possibility of such a complex anatomical scenario necessitates avoiding emergency delivery, thus requiring accurate birth planning. For this reason, elective caesarean section is the preferred mode of delivery [1, 7–9]. Consequently, multidisciplinary management involving obstetricians, urologists, urogynecologists, general and plastic surgeons, radiologists and neonatologists is fundamental.

### Patients and methods

A 32-year-old primigravida with a history of congenital BE and multiple surgical interventions was referred to our Department at 24 weeks of gestation. During childhood, the woman underwent repeated surgical procedures, including: ureteral reimplantation and bladder neck plastic surgery in early life; gastrocystoplasty and bladder enlargement with a gastric patch at the age of 9; plastic reconstruction of the external genitalia and clitoridoplasty the following year; bladder enlargement procedure at the age of 13, replacing the gastric patch with an ileo-colic segment, closing the bladder neck, and constructing a continent appendicostomy using the Mitrofanoff technique. Following these interventions, the patient began clean intermittent self-catheterization through the appendicostomy at intervals of approximately two hours. Over the subsequent years, due to renal calculi, genital anomalies, and an anterior abdominal wall hernia, she underwent additional procedures, including hernia repair using a biologic mesh.

**Fig. 1:**
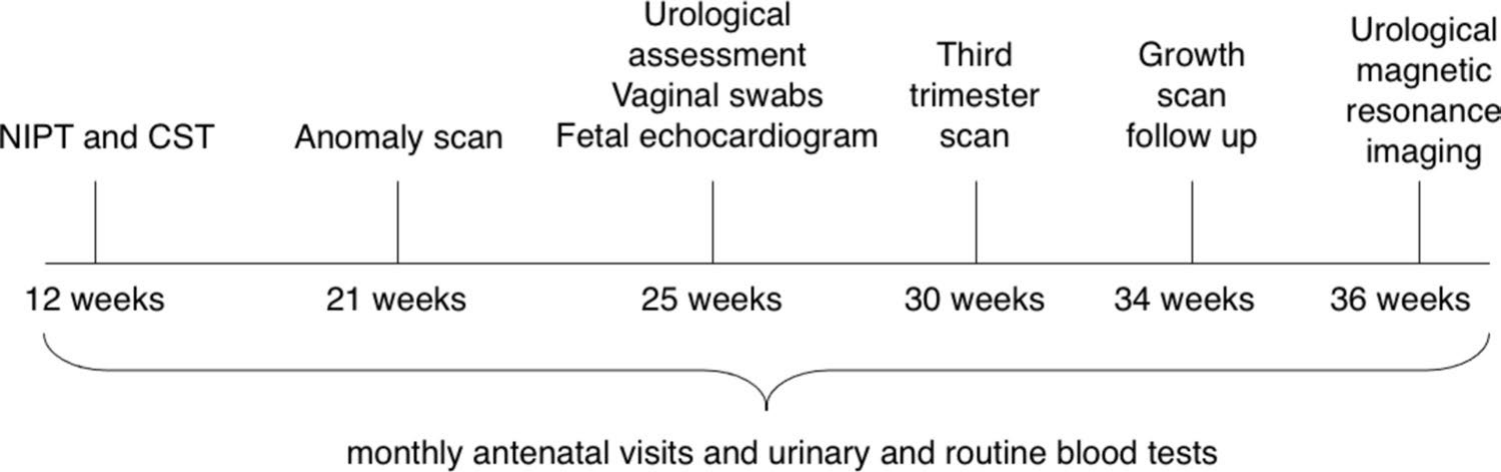
Timeline of antenatal care. *NIPT* non-invasive prenatal testing; *CST* combined screening testing

On initial obstetric evaluation, the abdominal wall surface presented multiple scars from previous surgeries and a Mitrofanoff appendicovesicostomy in the right iliac fossa. During pregnancy, the patient underwent routine antenatal care and preoperative anatomical assessment (Fig. 1). An elective caesarean section was planned at 37 weeks due to breech presentation. To ensure a safe abdominal wall incision, intraoperative ultrasound allowed the study of neobladder extension after the injection of 200cc of methylene blue (Bioindustria L.I.M.). Furthermore, a thorough review of the current literature was conducted to explore the relevant implications of this pathology and analyze the documented outcomes.

## Results

Pregnancy course was normal, with only urine cultures repeatedly positive for *Escherichia coli* (> 1 million CFU), which were treated with antibiotics according to antimicrobial susceptibility testing. Third trimester ultrasound scans revealed fetal growth restriction (FGR) with normal Dopplers and breech presentation. An elective caesarean section was planned at 37 weeks due to breech presentation. Given the patient’s history of recurrent urinary tract infections, which have been reported to be associated with a higher risk of preterm birth [10], and the presence of FGR, betamethasone was administered at 36 weeks to promote fetal lung maturation. This was in accordance with the local protocol, which recommends the administration of 12 mg in two intramuscular doses, one dose every 24 hours, over the seven days preceding the elective caesarean section in women at risk of late preterm birth. Preoperative anatomical assessment through urological magnetic resonance imaging revealed that the neobladder was medially located in the meso and hypogastric area, anteriorly compressed by the pregnant uterus. The neobladder showed a cranio-caudal extension of 17 cm, a lateral–lateral diameter of 26 cm, and  an anteroposterior diameter of 1.5 cm. The neobladder cranial margin projected towards the uterine fundus about 10 cm away.

Intraoperative ultrasound was used to assess the actual  anatomical extension of the neobladder, allowing for a safer surgical incision during the elective caesarean section. A midline laparotomy was performed. As showed in the video (Online Resource 1), once the peritoneal cavity was accessed, meticulous ileo-ileal and ileo-uterine adhesiolysis was safely performed. Once visceral adhesiolysis was completed, the distended neobladder appeared cranially displaced and adherent to the uterus. Consequently, the neobladder was carefully isolated. After visualizing the uterine fundus, a midline fundal anteroposterior incision was made. A cephalic extraction of a vital and healthy baby-weighing 2450 g (19^th^ centile, using the Italian Neonatal Study Charts growth charts), with 1- and 5-minute Apgar scores of 9 and 10, arterial pH 7.283, venous pH 7.325- was then performed. The fundal uterine incision facilitated the preservation of the neobladder. After delivery, the woman experienced an uncomplicated recovery, with no postoperative difficulties in catheterization through the stoma. She was discharged 3 days postpartum, with a follow-up visit scheduled one month later.

## Discussion

Obstetric management in women with congenital BE poses several challenges due to anatomical and physiological complexities. These women are at higher risk of maternal and fetal complications, including miscarriage, urinary tract infections, fetal malpresentation, premature delivery, urinary incontinence, and uterine prolapse. Multiple postoperative adhesions from previous surgeries may contribute to surgical risks during delivery, such as urinary tract damage or bowel perforation [1, 3, 5, 7, 11–13]. Given the complexity of BE, effective obstetric management requires extensive antenatal counselling, meticulous delivery planning, and a multidisciplinary approach in tertiary care centers [1, 9, 11]. Several authors underline the importance of involving urologists during pregnancy and delivery to prevent or manage potential urinary tract injuries [1, 3, 4, 7, 9, 13–16]. Furthermore, a comprehensive prenatal anatomical study is advisable to minimize complications. Some authors emphasize that performing an abdominal magnetic resonance imaging in the third trimester is crucial for determining the location of structures within the abdominal cavity [13, 17]. The mode of delivery remains a matter of debate. Most authors recommend avoiding emergency delivery due to the significant risk of surgical injury and neonatal complications resulting from difficult and prolonged access to the uterine cavity, advocating for planned caesarean section [1, 7–9]. However, cases of patients with multiple reconstructive surgeries who, despite the initial plan for caesarean section, successfully underwent spontaneous vaginal delivery are reported [3]. Additionally, the significantly higher incidence and progression of pelvic organ prolapse in women with congenital BE, regardless of parity, should be considered. The altered pelvic anatomy, combined with extensive adhesions from prior reconstructive surgeries, often limits the eligibility of these patients for conventional management of this gynecological condition [18, 19]. Consequently, caesarean delivery is strongly recommended to protect continence and prevent genital prolapse [4, 11, 20]. For unrepaired BE cases, Mandal et al. and Hanprasertpong et al. also recommend performing a caesarean delivery, with careful intrapartum and postpartum monitoring. In conclusion, according to the current literature, vaginal birth should be considered only when the pregnancy is entirely uncomplicated [1]. The choice of surgical incision during caesarean section is crucial to avoid major complications and preserve the integrity of reconstructed anatomical structures. Depending on the specific anatomy and surgical history, a midline or paramedian abdominal incision is usually utilized [11, 13]. Given that our patient already had multiple paramedian scars from previous surgical procedures, we opted for an individualized approach, performing a midline abdominal incision. We contribute to the existing literature by presenting a successful neobladder-saving caesarean section in a woman with congenital BE, providing a useful example for physicians facing such complex cases. Antenatal anatomical study played a pivotal role in delivery planning, with urological magnetic resonance imaging offering a safe and effective method for assessing the neobladder's position and dimensions. In addition, performing an intraoperative ultrasound further facilitated the precise localization of the neobladder’s extension and assured  the safe surgical approach  with low risk of complications. Overall, our experience highlights the importance of individualized management tailored to the anatomical complexity of each patient, alongside the value of multidisciplinary collaboration and preoperative planning in optimizing reproductive outcomes. Further research is warranted to develop specific guidelines and enhance pregnancy management for women with this rare and complex condition.

## Conclusions

Effective obstetric management in patients with BE requires a multidisciplinary approach and close collaboration among various expert specialists in a tertiary care center. Comprehensive delivery planning and antenatal anatomical studies are crucial for optimizing maternal and fetal outcomes. Our experience emphasize the importance of individualized management, tailored to the patient's anatomical peculiarity, to prevent intraoperative surgical complications and preserve neobladder and pelvic floor integrity.

## Supplementary Information

Below is the link to the electronic supplementary material.

Supplementary file1 (MP4 215914 kb)

## Data Availability

All relevant data are included in this article and its supplementary materials. Further information is available by contacting the corresponding author.
